# Mediterranean Diet and Melatonin: A Systematic Review

**DOI:** 10.3390/antiox12020264

**Published:** 2023-01-24

**Authors:** Elena Grao-Cruces, Juan Ramon Calvo, Maria Dolores Maldonado-Aibar, Maria del Carmen Millan-Linares, Sergio Montserrat-de la Paz

**Affiliations:** Department of Medical Biochemistry, Molecular Biology, and Immunology, School of Medicine, University of Seville, Avenida Sanchez Pizjuan s/n, 41009 Seville, Spain

**Keywords:** antioxidant, beer, circadian rhythm, nuts, tomato, olive oil, sleep, wine

## Abstract

The Mediterranean diet (MD) has beneficial effects on human health, which is evidenced by the observation of lower incidence rates of chronic diseases in Mediterranean countries. The MD dietary pattern is rich in antioxidants, such as melatonin, which is a hormone produced mainly by the pineal gland and controls several circadian rhythms. Additionally, melatonin is found in foods, such as fruit and vegetables. The purpose of this systematic review was to assess the melatonin content in Mediterranean foods and to evaluate the influence of the MD on melatonin levels in both humans and model organisms. A comprehensive search was conducted in four databases (PubMed, Scopus, Cochrane Library and Web of Science) and data were extracted. A total of 31 records were chosen. MD-related foods, such as tomatoes, olive oil, red wine, beer, nuts, and vegetables, showed high melatonin contents. The consumption of specific MD foods increases melatonin levels and improves the antioxidant status in plasma.

## 1. Introduction

The Mediterranean diet (MD) is a nutritional pattern with widely known health properties [1]. Traditionally, the MD is consumed in geographic areas where olives (*Olea europea* L.) and grapes (*Vitis vinifera* L.) are cultivated, and olive oil and wine are regularly produced and consumed [2,3]. Other features are associated with the benefits of the MD. The MD has been declared as part of the intangible heritage of humanity by UNESCO, but including other habits and factors of the Mediterranean countries, the weather and moderate exercise practice [4]. In terms of the nutritional pattern, the MD is balanced in calories and macro- and micronutrient intake. As essential components, the MD includes whole grains, fruits, vegetables, legumes, nuts, yogurt, fish, and white meat [5,6]. Minerals, vitamins, and other bioactive compounds are included in different foods of the MD, such as fruits, vegetables, and olive oil, among others [6,7,8]. The MD dietary habits are correlated with a lower incidence of cancer [9,10], cardiovascular [11,12] and neurodegenerative diseases [13,14,15], when compared to industrialized countries’ dietary patterns [16].

From a phytochemical point of view, the MD is rich in phenylpropanoids, isoprenoids, and alkaloids [6,17], compounds with high antioxidant activity. In fact, the antioxidant properties of the bioactive compounds included in the MD are some of the major contributors to the health properties of the MD [18]. For example, polyphenols have been widely studied in terms of the antioxidant properties of the MD [19,20,21,22]. On the other hand, melatonin has been recently characterized as a phytochemical element in MD foods, which increases the MD’s health potential [23,24]. 

Melatonin is a hormone that is biosynthesized from tryptophan in four well-defined intracellular steps [25]. Melatonin synthesis is regulated by the light/dark cycle and the enzymes involved in the process are expressed during the night, because the light inhibits their expression [26]. It is involved in different plant functions, such as plant growth regulation, delaying flowering delay, photosynthetic system protection, oxidative stress damage protection, and biotic and abiotic stresses [27,28,29]. Melatonin was first discovered as a secretory product of the pineal gland. It is the main chronobiotic hormone that regulates the circadian rhythms [30] and seasonal changes in vertebrate physiology via its daily nocturnal increase in blood [31,32]. However, subsequent studies showed that melatonin is present in bacteria, unicellular eukaryotic organisms, invertebrates and vertebrates, algae, plants, and fungi, and is found in various edibles, such as vegetables, fruit, herbs, and seeds [33,34]. In mammals, melatonin is synthesized in many tissues and organs [35,36,37,38,39,40], and melatonin shows remarkable functional versatility, exhibiting antioxidant [41], oncostatic [42], antiaging [43], and immunomodulatory [44] effects. The antioxidant functions of melatonin have been widely described. Melatonin is a free radical scavenger; it directly scavenges reactive oxygen species (ROS) and nitrogen-based species with even more effectivity than vitamin E [45,46,47,48,49]. Additionally, melatonin induces the activity of antioxidant enzymes, such as glutathione reductase and peroxidase [50,51].

The MD is a nutritional pattern rich in many antioxidants and bioactive compounds; therefore, the identification of melatonin in edibles and the multiple functions of melatonin are an interesting perspective. The aim of this systematic review was to assess the melatonin content in Mediterranean foods and to evaluate the influence of the MD on melatonin levels in both humans and model organisms.

## 2. Materials and Methods

This systematic review was conducted following the Preferred Reporting Items for Systematic reviews and Meta-analysis (PRISMA) [52,53]. This review was registered on the International Prospective Register of Systematic Reviews (PROSPERO, registration No. CRD42022332235). 

### 2.1. Searching Strategy

A comprehensive search was conducted in four databases (PubMed, Scopus, Web of Science and Cochrane Library), searching all years of records up until February 2022. The language restriction was English and Spanish. The search terms were categorized into the following three key concepts: study population, MD (MD-related foods and intervention) and melatonin levels. The specific terms used were “adult” OR “middle-aged” OR “young” OR “m?n” OR “wom?n” OR “food” OR “plant” AND “mediterranean diet” OR “Olive Oil” OR “grape” OR “tomato” OR “nuts” OR “legume” OR “cereal” AND “melatonin”, respectively. In addition, the reference lists of the elected articles were manually searched for relevant publications. To choose which MD-related foods were specifically included in the research algorithm, foods previously described to have melatonin content were used, based on the work of Meng et al. [54]. In regard to the MD, the foods were selected according to Schwingshackl et al. [6].

### 2.2. Selection Criteria

The published studies in this review were required to adhere to the following criteria: (1) they must be original research; (2) human adult or animal studies or edible food studies; (3) MD or MD-related food interventions or analyses; (4) must include melatonin measurements.

### 2.3. Data Extraction and Reliability

The PRISMA recommendations were followed [52,53]. First, in order to identify relevant articles, the titles were screened and abstracts were analyzed. Then, the selected articles were fully reviewed for eligibility by two independent researchers.

## 3. Results

### 3.1. Search and Selection of Studies

**Figure 1:**
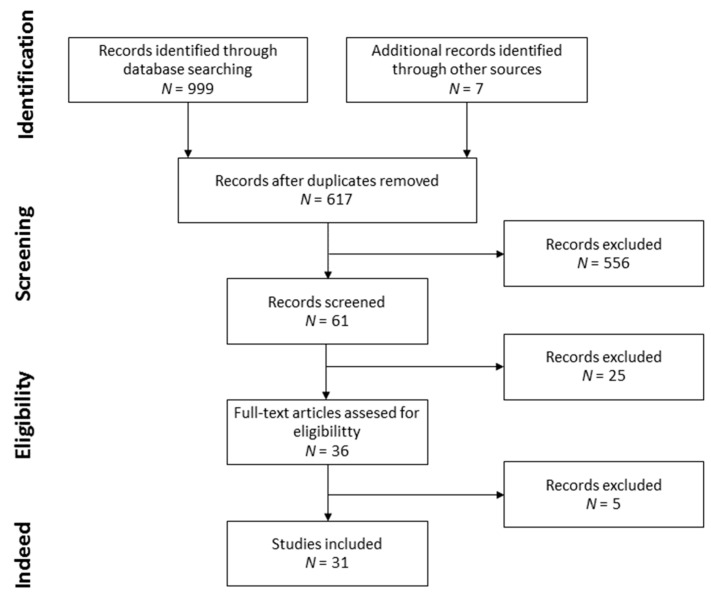
Flow diagram of record selection.

The searching and selection strategies are detailed in Figure 1. A total of 999 and 7 records were identified through database searching and the search of the reference lists of the retrieved articles, respectively. Duplicates were removed, leaving a set of 617 records, from which 36 records were screened. Finally, 5 records were excluded for not following the selection criteria and 31 records were selected. The records were divided into the following two different categories: foods from the MD with melatonin [55,56,57,58,59,60,61,62,63,64,65,66,67,68,69,70,71,72,73,74,75,76,77,78,79,80] and influence of the MD on melatonin levels [60,72,79,80,81,82,83,84,85,86].

### 3.2. Melatonin in Mediterranean Diet Foods

#### 3.2.1. Melatonin Content in Fruit and Vegetables

Melatonin is present in different MD-associated foods and drinks, with grapes and olive oil being the first two products of the MD in which melatonin was detected (Table 1). Wine is a grape by-product drink associated with the nutritional pattern of the MD and is rich in phytochemicals, such as polyphenols and melatonin. Red wines are richer in melatonin (7.44–0.24 ng/mL) compared to white wines (3.93–0.16 ng/mL), which is similar to grapes [55,56,58]. However, Rodriguez-Naranjo et al. showed that the melatonin content of different wines from Spain was between 423.01 and 74.14 ng/mL, with no differences being observed between white and red wines [57]. It is important to consider that different factors, such as environmental factors, agricultural practices, vintage, and wine-making procedures, may influence the melatonin levels in wine. For example, the melatonin content was different among different brands that produce the same wine [56]. Additionally, Albana grape-derived wine showed a lower content of melatonin compared to the Albana grape itself (the melatonin level of which is 1.2 ng/g), supporting the hypothesis that part of melatonin is lost during the wine-making process [55]. Beer is other typical beverage in the MD; however, the melatonin content in beer was lower when compared to wines [60]. 

Olive oil is a by-product of olive that is known worldwide for its anti-inflammatory and antioxidant properties, while virgin olive oil is the main oil used in the MD. De la Puerta et al. measured the melatonin content of different virgin olive oils and reported values that ranged between 71 and 119 pg/mL, with the highest amount of melatonin being detected in virgin olive oils produced via the cold process. Additionally, the refinement process reduces the melatonin content in olive oil, even though refined olive oil showed a higher content of melatonin than sunflower oil [61].

The highest melatonin content was detected in fruit and vegetables (Table 2), but the melatonin quantity in the same fruit varies depending on the harvest conditions, such as the time of day, year, maturity, and variety. For example, tomato is one of most consumed fruit in the MD and its melatonin content has been widely studied, with wide differences being observed between varieties. Stürtz et al. studied different tomato varieties, including *Lycopersicon esculentum* from Spain, and the highest content of melatonin was detected in the Raf and Bond varieties, with 50 and 25 ng/g, respectively [62]. It is important to highlight that Dubbels et al. [64] and Pape et al. [63] measured the melatonin content of other varieties of *Lycopersicon esculentum* grown in Germany, showing a 10–100-times lower content when compared with that described by Stürtz et al. Pape et al. measured melatonin content using ELISA and HPLC-PD and different organic solvents to extract melatonin, and the melatonin content was between 0.5984 and 1.0685 ng/g [63], which was similar to the findings of Dubbles et al. [64]. Despite the fact that Pape et al. [63] and Stürtz et al. [62] used the same tomato species (*L. esculentum*) and HPLC to measure the melatonin content, the solvents for the extraction and the harvest processes were different. Additionally, Stürtz et al. studied the melatonin content in *L. esculentum* and found that it differed within the same tomato variety because of the different harvests [62]. 

In regard to *Solanum lycopersicum*, the melatonin content found by all authors [66,67,68] was lower compared to that of *Lycopersicum esculentum* determined by Stürtz et al. [62]. However, Spanish *Solanum lycopersicum* [68] showed the highest melatonin content, when compared to the same species grown in other countries [66,67].

#### 3.2.2. Melatonin Content in Nuts, Legumes, and Animal-Derived Products

Different types of nuts, including almonds, pistachios, nuts, and chestnuts, which grow in Mediterranean countries, are included in the nutritional pattern of the MD and have approximately 1 ng/g of melatonin (Table 3). The nut variety influences melatonin levels in nuts, with different varieties of almonds (*Prunus dulcis* L.) and pistachios (*Pistacia vera* L.) showing melatonin content values between 600–2000 pg/g and 1000–12,000 pg/g, respectively [73,74]. Verde et al. measured melatonin in almonds and pistachios by HPLC-FLD after extraction with a combination of organic solvents and found a similar quantity of melatonin to Paroni et al., who measured melatonin content by LC-MS/MS after SPE extraction in both types of nuts. However, Bronte DOP pistachio and cv. Palo almond showed higher contents of melatonin when compared to other varieties. These differences are greater when comparing pistachio varieties [74]. It is important to highlight that Oladi et al. determined the melatonin content in four different varieties of pistachio from Iran, which was 1000–10,000-times higher than that of other nuts and pistachio varieties [75]. In contrast, Manchester et al. measured the melatonin content in almonds derived from another species referred to as *Prunus amygdalus* [76], showing 10-times higher levels compared to that of *Prunus Dulcis*. Additionally, it is important to consider that nuts can be eaten raw or roasted, and the processing of foods influences melatonin bioavailability. In fact, it was reported that roasting decreased melatonin content in most nuts, while this was increased in roasted peanuts [73].

Legumes and grains are one of the main components of the MD and are very rich in melatonin, in contrast with animal-derived products, such as meat, fish and eggs, in which melatonin content is very low (Table 4). Lentils showed the highest amount of melatonin, which was doubled in sprouts, compared to raw lentils [80]. However, comparison with other studies cannot be made, because there are no multiple records regarding the melatonin content in the same legume.

### 3.3. Melatonin Levels and Mediterranean Diet

Despite the fact that different foods of the MD contain melatonin, the influence of their intake is not clear. All the records that show data of melatonin levels after the intake of the MD are shown in Table 5. The consumption of melatonin-rich foods, such as fruit and legumes, increased melatonin levels in humans and animal models. However, the available data are not comparable, because different foods were used in all the records of the different study populations. 

For example, rats fed with 3 g of walnuts (*Juglans regia* L.), which contain about 10.5 ng of melatonin, showed increased serum melatonin concentrations from 11.5 to 38.0 pg/mL, and their serum total antioxidant capacity (TAS) also increased [72]. In contrast, in humans, the serum melatonin concentration was significantly higher 1 h after the intake of 100 mL of red wine [82]. In addition, it has been reported that the moderate consumption of beer (330 mL and 660 mL for women and men, respectively) increased both melatonin and TAS of human serum after 45 min of beer ingestion [60].

## 4. Discussion

The MD is demonstrated to have benefits for human health, because of the nutritious quality of its foods. The MD nutritional pattern includes a high consumption of fruit and vegetables, mono and polyunsaturated fats, and proteins, within products rich in micronutrients, with antioxidant and immunomodulatory effects. The melatonin content in edibles has been widely studied; however, the existing evidence for some products is not conclusive. The factors that influence the differences in melatonin content include melatonin extraction and measurement techniques, species or variety of the food, growing and harvest conditions and food processing; however, their level of influence remains unknown. 

There are different protocols for melatonin extraction and quantification. Organic solvents, in combination with different purification steps, have been predominantly used for melatonin extraction [87]. Additionally, the organic solvents and their proportion used are important for melatonin extraction, with the most commonly used solvents being methanol and chloroform. In regard to melatonin quantification, techniques such as radioimmunoassay, ELISA, HPLC and mass spectrometry were used. Radioimmunoassay and ELISA are less specific techniques than HPLC for measurements in plant material because there could be cross-reactivity with other plant metabolites. HPLC techniques are highly sensitive, accurate and versatile and can be used with very different biological matrices, and HPLC coupled with fluorescence detection is the most used technique in the records studied. Mass spectrometry has been also used in various records due to its high sensitivity, but mass spectrometry techniques usually have problems with plant matrices. Some authors validated their methodology and performed studies under diverse conditions and following distinct techniques [55,56,58,73,74,75]. The results among all the records showed differences in melatonin quantity depending on the methodology followed, but the variability shown by different methodologies does not explain the variabilities found by different authors for the same product. 

In contrast, the level of influence of other factors on melatonin content is not clear. There is ample research about the post-harvest exogenous melatonin effects in fruits and vegetables [88,89,90], and some studies focus on the environmental effects on melatonin production in fruits and vegetables. For example, the growing conditions play a crucial role in the melatonin content of foods. For example, Tan et al. reported that sunlight exposure is a conditioning factor, because sunlight stimulates melatonin synthesis in plants [91]. Additionally, there is evidence that suggests that Mediterranean plants, which grow under high sunlight exposure, have higher melatonin content, compared to the same species living under lower sunlight exposure in other locations. In addition, it is not clear whether the ripening process modulates the melatonin content in fruit. For example, the melatonin amount in grape berries decreases with ripening [92], but other studies have reported constant melatonin content during ripening [93]. In addition, melatonin is described to have a circadian rhythm in fruit, showing increased levels during the day; therefore, the moment of the harvest would determine the melatonin content [94]. 

There is extensive evidence in relation to the effects of melatonin supplementation [95]. On the contrary, little has been reported about the effects of melatonin consumption via foods with high melatonin content. The consumption of foods rich in melatonin increased melatonin levels in blood; however, the mechanism has not yet been described. The increase in melatonin in blood could be due to a retention in the fall of melatonin content during the day or to an increase in melatonin synthesis. Additionally, it is important to consider that melatonin is a hormone that follows a circadian rhythm in humans and animals. Therefore, the time when melatonin is ingested, and the time of the measurement are crucial factors. Only one of the human studies carried out an intervention with melatonin-rich foods before bed time and they found an increase in blood melatonin and an improvement in sleep quality after tomato consumption [81]. Nevertheless, the effects on sleep quality could be due to other compounds in the tomato. The remaining human studies monitored the intake of food after overnight fasting and found an increase om melatonin and its derivatives. An improvement of sleep quality was not evaluated by the authors, since melatonin is rapidly (30–50 min) degraded by different cytochromes [96], so an early intake of melatonin probably would not have an effect on sleep quality. 

The antioxidant effects of intrinsic food melatonin consumption have been studied in three different articles included in this review [60,72,79], and the authors associated an increase in the antioxidant capacity with the melatonin intake within the foods being evaluated, including fruits, nuts and beer. For example, Maldonado et al. 2013 suggested that higher TAS was associated with higher melatonin concentration in beer [60]. However, other components of beer have not been evaluated and this includes the work of Reiter et al. 2005 and Sae-Teaw et al. 2013, when they associated nut consumption with TAS [72]. Serum antioxidant capacity was measured with two different protocols by Sae-Teaw et al. in 2013 after the consumption of different fruits, and the increase in the antioxidant capacity of serum correlated with melatonin increase in serum [79]. Melatonin has been broadly established as a potent antioxidant molecule [45,46,47,48,49,50,51], so the increases in TAS and antioxidant capacity after nut, beer and fruit intake could be associated with melatonin, but other food components could also affect TAS, such as polyphenols, which are abundant in beer [97,98], nuts [99,100,101] and fruits [102,103], also increase TAS [104,105].

In regard to animal studies, only one [83] performed a long-term study, in which an oil extracted from nuts resulted in an increase in blood melatonin levels, an effect that was enhanced when this oil was combined with unsaturated fatty acids. In contrast, all the other studies reported a one-time intervention, in which an increase in melatonin was detected after the intake of foods with high melatonin content. However, the model animals used were different in all the records; therefore, a measurement of the effect could not be determined. Additionally, the studies did not analyze the same food; therefore, there is only a single piece of evidence for each food item studied. 

Despite all the data analyzed in this review about the increase in melatonin levels after the intake of melatonin-rich products, it is important to highlight that an increase in melatonin is not consistent with the melatonin content of the foods. Therefore, the effects of the consumption of certain products are not only due to their melatonin content, but rather the product’s matrix and other components may play a role in melatonin metabolism that enhances the effect of melatonin intake. 

In conclusion, the melatonin content in foods is not a constant parameter; however, tomato, wine, beer, nuts, and olive oil are MD foods with melatonin and specially, specific varieties of these foods have high contents of melatonin. The punctual or periodical consumption of these foods could contribute to increased blood melatonin levels and could have an impact on sleep quality and antioxidant status. 

## Figures and Tables

**Table 1:** Melatonin content in grapes, wine, beer, and oils of the MD.

	Variety	Melatonin (ng/g; ng/mL)	Melatonin Extraction	Melatonin Measure	Reference
Grape	Sangiovese grape	1.5	MEPS with methanol	HPLC-FLD	[55]
	Albana grape	1.2			
	Albana must	1.1			
	Albana grape juice	0.5			
White wine	Albana	0.6	MEPS with methanol	HPLC-FLD	[55]
	Multivarietal	0.63	DLLME with acetonitrile, chloroform and NaCl	HPLC-FLD	[56]
	Moscatel Graudo	3.93			
	Palomino fino	390.82	SPE with methanol and water	LC-ESI-MS/MS	[57]
	Sauvignon blanc	0.32	Methanol and sonication	Electrochromatography	[58]
	Chardonnay	0.16			
Red wine	Malbec	0.24	Methanol and sonication	Electrochromatography	[58]
	Cabernet Sauvignon	0.23	SPE with methanol and water	ELISA	[59]
	Jaen tinto	0.16			
	Merlot	0.21			
	Palomino negro	0.28			
	Petit verlot	0.22			
	Prieto picudo	0.19			
	Syrah	0.22			
	Tempranillo	0.14			
	Castelao	7.44	DLLME with acetonitrile, chloroform and NaCl	HPLC-FLD [28]	[56]
	Syrah	4.29–2.61			
	Trincadeira	1.90			
	Aragonez	4.27			
	Touriga Nacional	2.71–2.81			
	Touriga Franca	1.05			
	Alicante Bouschet	1.92			
	Cabernet Sauvignon	2.85–3.06			
	Syrah	423.01	Methanol and sonication	LC-ESI-MS/MS	[57]
	Cabernet Sauvignon	74.13			
	Merlot	245.46			
	Tempranillo	306.86			
	Tintilla de Rota	322.68			
Beer	Volt-Damm	0.1697	NS	ELISA	[60]
	Murphy’s	0.1427			
	Mahou Negra	0.1386			
	Amstel	0.1281			
	Coronita	0.1276			
	Budweisser	0.1198			
	Guiness	0.1181			
	Cruzcampo	0.1119			
	Calsberg	0.1044			
	Mahou 5 estrellas	0.1019			
	Heineken	0.0981			
	San Miguel Sp	0.0976			
	Mahou Clasica	0.0846			
	Laiker Sin	0.0686			
	San Miguel 0.0	0.0616			
	Buckler Sin	0.0616			
	Kaliber Sin	0.0527			
	Buckler 0.0	0.0518			
Olive oil	D.O Sierra Magina	0.107	Liquid–liquid extraction with methanol and chloroform	ELISA	[61]
	D.O. Siurana	0.095			
	D.O. Bajo Aragon	0.071			
	D.O Montes de Toledo	0.108			
	D.O. Baena	0.119			
	D.O. Sierra de Segura	0.089			
	D.O. Les Garrigues	0.098			
	D.O. Toscano	0.109			
	Refined olive oil	0.053–0.075			

DLLME, dispersive liquid–liquid microextraction; ELISA, enzyme-linked immunoassay; HPLC-FLD, high-precision liquid chromatography with fluorescence detection; LC-ESI-MS/MS, liquid chromatography electrospray ionization tandem mass spectrometry; MEPS, microextraction by packed sorbent or packaged syringe.

**Table 2:** Melatonin content in fruit and vegetables.

Food			Melatonin ng/g		Melatonin Extraction	Melatonin Measure	Reference
	Species	Variety					
Tomato	*L. esculentum*	Bond	23.87		Methanol and water	LC-FL	[62]
		Borsalina	8.2				
		Catalina	4.1				
		Gordal	17.10				
		Lucinda	4.45				
		Marbone	18.13–114.5				
		Myriade	8.0				
		Pitenza	14.0–14.2				
		Santonio	ND				
		Perlino	ND				
		Platero	13.6				
		Raf	50.1				
		NS	0.9821		Na_2_CO_3_ ether	HPLC-PD	[63]
			1.0685			ELISA	[63]
			0.6169		SPE with PCA	HPLC-PD	[63]
			0.5984			ELISA	[63]
			0.8963		SPE with acetone	HPLC-PD	[63]
			0.9363			ELISA	[63]
		Mill. Cultivar Sweet 100	0.506		Na_2_CO_3_ and diethyl ether	Radioimmunoassay	[64]
		Mill. Cultivar Rutgers California supreme	0.166				
	NS	Crystal	0.029		Initial extraction with ethyl acetate, followed by SPE with methanol and water	UHPLC-q-Orbitrap MS	[65]
		Raspberry	0.0323				
		Oxheart	0.0151				
		GiantPaste	0.0493				
		Beorange	0.0232				
		Hurma	0.0181				
		Lemon	0.0299				
		Raspberry	0.0492				
		Yellow plum	0.0324				
		Azoycka	<0.01				
		Giant	<0.01				
		Better Boy	0.0174				
		Ox’s Forehead	0.0418				
		Yellow Plum	0.0157				
		Roma	0.0594				
		Mini Plums	0.0219				
		Cherry	0.0788				
		Cherry Red	0.0149				
		Kiwi	0.024				
		Green Pepper	0.0141				
		Black Moor	0.0242				
		Black Broken Heart	0.0141				
		Kumato	0.0242				
		Mojito	0.0209				
		Acrobat Tom	0.0453				
		Beauty Lottringa	0.0138				
		Raspberry	0.0246				
		Plum	0.0251				
	*Solanum* *lycopersicum*	Cerasiforme	1.618		SPE and methanol	HPLC-FLD	[66]
		Micro-Tom	0.001–0.003		SPE with acetone and methanol	ELISA	[67]
		Ciliegia	0.64		Methanol	UHPLC-QqQ-MS/MS	[68]
		Isis	1.5				
		Jack	1.5				
		Prico	3				
		Jesus	4				
		NKT 072	6				
		Optima	14.77				
	*L. pimpinellifolium*		0.112		Na_2_CO_3_ and diethyl ether	Radioimmunoassay	[64]
Sweet cherry	*Prunus avium* L.		60		Methanol and ultrasonication	HPLC-FLD	[69]
Strawberries	*Fragaria ananassa*	Camarosa	1.4–5.58		SPE with methanol and acetone	LC-FL	[62]
		Candonga	2.1–5.5				
		Festival	3.28–11.26				
		Primoris	4.2–8.5				
	*Fragaria magna*		0.136		Na_2_CO_3_ and diethyl ether	GC/MS	[70]
Banana	*Musa ensete*		0.655		Na_2_CO_3_ and diethyl ether	GC/MS	[70]
	*Musa sapitentum*		0.41		Diethyl ether	Radioimmuno assay	[64]
			1.741		SPE with methanol	HPLC-FLD	[66]
Pineapple	*Ananas comosus*		1.693		SPE with methanol	HPLC-FLD	[66]
			0.278		Na_2_CO_3_ and diethyl ether	GC/MS	[70]
Apple	*Malus domestia*		0.161		Na_2_CO_3_ and diethyl ether	GC/MS	[70]
Pomegranate	*Punica granatum*		0.16.8		Na_2_CO_3_ and diethyl ether	GC/MS	[70]
Watermelon	*Cirtullus lanatus*		ND		SPE with methanol	HPLC-FLD	[66]
Orange	*Citrus sinensis*		1.704		SPE with methanol	HPLC-FLD	[66]
Bell pepper	NS	Green	0.0255–0.5214		Methanol and ethyl acetate	HPLC-FLD	[71]
		Orange	0.045– 0.0495		Ethyl acetate	HPLC-FLD	[71]
			0.5811		Methanol	HPLC-FLD	[71]
		Red	0.0243–0.0664		Ethyl acetate	HPLC-FLD	[71]
			0.1795		Methanol	HPLC-FLD	[71]
	*C. anuum*	Barranca	Green	2	Methanol	UHPLC-QqQ-MS/MS	[68]
			Red	4			
		Derio	Green	3.5			
			Red	6			
		Velero	Green	10			
			Red	4			
		F26	Green	2			
			Red	11			
		NC9	Green	2.5			
			Red	4			
Cucumber	NS		0.009		Diethyl ether	Radioimmuno assay	[64]
	*Cucumis sativus*		0.592		Na_2_CO_3_ and diethyl ether	GS/MS	[70]
Onion	*Allium cepa*		29.9		Na_2_CO_3_ and diethyl ether	GS/MS	[70]
Garlic	*Allium sativum*		58.7		Na_2_CO_3_ and diethyl ether	GS/MS	[70]
Cabbage	*Brassica oleraceae*	Capitata	30.9		Na_2_CO_3_ and diethyl ether	GS/MS	[70]
Cauliflower	*Brassica oleraceae*	Botrytis	82.4		Na_2_CO_3_ and diethyl ether	GS/MS	[70]
Turnip	*Brassica rapa*		50.1		Na_2_CO_3_ and diethyl ether	GS/MS	[70]
Carrot	*Daucus carota*		49.2		Na_2_CO_3_ and diethyl ether	GS/MS	[70]
Barley	*Hordeum vulgare*		87.3		Na_2_CO_3_ and diethyl ether	GS/MS	[70]
Radish	*Raphnus sativus*		75.8		Na_2_CO_3_ and diethyl ether	GS/MS	[70]
Beetroot			0.0001		Diethyl ether	Radioimmuno assay	[64]
Potato			ND		Diethyl ether	Radioimmuno assay	[64]
			ND		Na_2_CO_3_ and diethyl ether	GC/MS	[70]

ELISA, enzyme-linked immunoassay; GC/MS, gas chromatography–mass spectrometry; LC-FL, liquid chromatography with fluorescence detection; PCA; SPE, solid-phase extraction; UHPLC, ultra-high-performance liquid chromatography; ND, not detected; NS, not specified.

**Table 3:** Melatonin content in nuts and seeds.

Food			Melatonin pg/g	Melatonin Extraction	Melatonin Measure	Reference
Species		Variety				
Walnut *Junglans regia* L.		Bulk	3500	Liquid–liquid extraction with methanol and chloroform	HPLC	[72]
		Pizarro	1191	First extraction with hexane–methanol–water, followed by SPE with acetonitrile and 0.1 % formic acid	HPLC-FLD	[73]
		Franquette	1600			
		Hartley	3301			
		Native	2000			
Pistachio *Pistacia vera* L.		NS	1000	First extraction with hexane–methanol–water, followed by SPE with acetonitrile and 0.1 % formic acid	HPLC-FLD	[73]
		Bronte DOP	12,000	First extraction with ethanol, followed by SPE with water and methanol	LC-MS/MS	[74]
		Noberasco	1000			
		Italy	1000			
		Italy	1000			
		Ahmed Aghaes	233,000,000	UASLE with methanol	GC/MS	[75]
			230,700,000		Spectrofluorometry	[75]
		Akbari	226,900,000		GC/MS	[75]
			229,200,000		Spectrofluorometry	[75]
		Karle Qouchi	231,400,000		GC/MS	[75]
			229,100,000		Spectrofluorometry	[75]
		Fandoghi	130,700,000		GC/MS	[75]
			228,400,000		Spectrofluorometry	[75]
Hazelnut *Corylus avellana* L.			350	First extraction with hexane–methanol–water, followed by SPE with acetonitrile and 0.1 % formic acid	HPLC-FLD	[73]
Almond	*Prunus dulcis* L.	NS	1100	First extraction with hexane–methanol–water, followed by SPE with acetonitrile and 0.1 % formic acid	HPLC-FLD	[73]
		cv. Palo	2000	First extraction with ethanol, followed by SPE with water and methanol	LC-MS/MS	[74]
		cv. Collepasso	600			
		cv. Barletta	1500			
		cv. Cassano	1300			
		cv. Minervino	600			
	*Prunus amygdalus*		39,000	Ethanol	HPLC	[76]
Peanut *Arachis hypogaea* L.			83	First extraction with hexane–methanol–water, followed by SPE with acetonitrile and 0.1 % formic acid	HPLC-FLD	[73]
			39,430	Organic solvents and sonication	HPLC-FD	[77]
Pine nuts *Pinus pinea* L.			1000	First extraction with hexane–methanol–water, followed by SPE with acetonitrile and 0.1 % formic acid	HPLC-FLD	[73]
Pumpkin seed *Cucurbita pepo* L.			500	First extraction with hexane–methanol–water, followed by SPE with acetonitrile and 0.1 % formic acid	HPLC-FLD	[73]
Sunflower *Helianthus annuus* L.		Seed	500	First extraction with hexane–methanol–water, followed by SPE with acetonitrile and 0.1 % formic acid	HPLC-FLD	[73]
		NS	67,450	First extraction with ethanol, followed by water and dichloromethane	HPLC-FD	[77]
Cashew nut *Anacardium occidentale* L.			200 (roasted)	First extraction with hexane–methanol–water, followed by SPE with acetonitrile and 0.1 % formic acid	HPLC-FLD	[73]
Macadamia nut *Macadamia intergriogolia*		Maiden y Betche	300	First extraction with hexane–methanol–water, followed by SPE with acetonitrile and 0.1 % formic acid	HPLC-FLD	[73]
Brazil nut *Bertholleita excels*		Humb and Bonpl	100	First extraction with hexane–methanol–water, followed by SPE with acetonitrile and 0.1 % formic acid	HPLC-FLD	[73]
Chestnut *Castanea sativa*		Miller	1417	First extraction with hexane–methanol–water, followed by SPE with acetonitrile and 0.1 % formic acid	HPLC-FLD	[73]

GC/MS, gas chromatography–mass spectrometry; HPLC, high-performance liquid chromatography; SPE, solid-phase extraction; NS, not specified.

**Table 4:** Melatonin content in meat, fish, egg, and oils.

Food		Melatonin ng/g	Melatonin Extraction	Melatonin Measure	Reference
Chicken		2.3	First extraction with PB and chloroform, followed by SPE with methanol	HPLC	[78]
Lamb		1.6	First extraction with PB and chloroform, followed by SPE with methanol	HPLC	[78]
Beef		2.1	First extraction with PB and chloroform, followed by SPE with methanol	HPLC	[78]
Pork		2.5	First extraction with PB and chloroform, followed by SPE with methanol	HPLC	[78]
Salmon		3.7	First extraction with PB and chloroform, followed by SPE with methanol	HPLC	[78]
Eggs		6.1	First extraction with PB and chloroform, followed by SPE with methanol	HPLC	[78]
Corn	Whole yellow corn	1.3	First extraction with PB and chloroform, followed by SPE with methanol	HPLC	[78]
	Corn germ meal	1.0			
	Waxy corn	2.704	First extraction with ethanol, followed by water and dichloromethane	HPLC-FD	[79]
	Waxy berry corn	2.797			
Red bean		54.79	First extraction with ethanol, followed by water and dichloromethane	HPLC-FD	[79]
Soybean		56.49	First extraction with ethanol, followed by water and dichloromethane	HPLC-FD	[79]
Lentils	Raw	460	Ethanol and sonication	HPLC-ESI-MS/MS	[80]
	Sprouts	1020			

HPLC, high-performance liquid chromatography; HPLC-ESI-MS/MS, high-performance liquid chromatography/electrospray ionization tandem mass spectrometry; HPLC-FD, high-performance liquid chromatography with fluorescence detection; PB; phosphate buffer; SPE, solid-phase extraction.

**Table 5:** Characteristics of the interventional studies.

Scheme	Sample	N	Age	Food	Design	Duration	Melatonin-Related Measure	Outcome
Yang [81]	Obese post-menopausal women	36	50–70 years old	Tomato	Intake of tomato before bed time	8 weeks	ELISA of aMT6 in urine	Beefsteak tomato showed a high quantity of MT and produced an increase in the MT levels and an improvement of sleep quality
Varoni [82]	Healthy volunteers	12	20–30 years old	Red wine enriched with melatonin	Morning wine intake after 12 h of fasting	1 intake	HPLC-LC/MS of melatonin in blood and saliva	MT-enriched wine and wine increased MT levels The matrix of the wine could contribute to the increased MT levels It counteracted the decrease in MT in the morning
Sae-Teaw [79]	Healthy male volunteers	12	18–25 years old	Pineapple Orange Banana	Morning fruit or fruit juice intake after 12 of fasting	1 intake	ELISA for blood melatonin	Fruit and fruit juice consumption increased blood MT and antioxidant capacity
Johns [83]	Healthy volunteers (both genders)	30	18–25 years old	Banana Pineapple Orange Papaya Makmao Mango	Morning intake after 12 h of fasting	1 intake	ELISA for a6MT in urine	Fruit consumption increased MT levels
Maldonado [60]	Healthy volunteers (both genders)	7	20–30 years old	Beer	Morning intake after 12 h of fasting	1 intake	ELISA and HPLC for blood melatonin	Beer consumption increased serological melatonin content compared to baseline
Ribas-Latre [84]	Male rats	24	11 weeks old	Grape seed proanthocyanidin extract	Intake of the diet	1 intake	ELISA for blood melatonin	Proanthocyanidin consumption increased MT levels and regulated the expression of circadian rhythm-related genes
Rebollo-Hernanz [80]	Male rats	48	6 weeks old	Lentis sprouts	Intake after 12 h of fasting	1 intake	ELISA for a6MT in urine and melatonin in blood	Lentils sprouts increased blood MT levels more than synthetic MT Lentils sprouts increased antioxidant capacity
Reiter [72]	Male rats	16	8 weeks old	Bulk walnuts	Intake after 12 h of fasting	1 intake	Radioimmunoassay for melatonin in blood	MT content was 20-times higher in the group fed with walnuts
Garcia [85]	Murine mammary adenocarcinoma model	60	16 weeks old	Nut oil	Intake of the diet	3 months	HPLC for blood melatonin	Blood MT was higher after nut consumption
Aguilera [86]	Rat	32	6 weeks old	Bean sprouts	Intake after 12 and 24 h of fasting	1 intake	ELISA for blood melatonin and a6MT in urine	Bean sprouts increased blood MT levels more than synthetic MT

ELISA, enzyme-linked immunoassay; HPLC, high-performance liquid chromatography; HPLC-LC/MS, high-performance liquid chromatography–liquid chromatography mass spectrometry; NS, not significant.

## Data Availability

Data sharing is not applicable to this article, as no datasets were generated or analyzed during the current study.
